# Myoferlin, a multifunctional protein in normal cells, has novel and key roles in various cancers

**DOI:** 10.1111/jcmm.14648

**Published:** 2019-09-01

**Authors:** Wei Zhu, Bolun Zhou, Chenxuan Zhao, Zhengqing Ba, Hongjuan Xu, Xuejun Yan, Weidong Liu, Bin Zhu, Lei Wang, Caiping Ren

**Affiliations:** ^1^ The NHC Key Laboratory of Carcinogenesis and The Key Laboratory of Carcinogenesis and Cancer Invasion of the Chinese Ministry of Education Xiangya Hospital Central South University Changsha Hunan China; ^2^ Cancer Research Institute Collaborative Innovation Center for Cancer Medicine School of Basic Medical Science Central South University Changsha Hunan China

**Keywords:** angiogenesis, cancer, metastasis, myoferlin, therapeutic target, vesicle trafficking

## Abstract

Myoferlin, a protein of the ferlin family, has seven C2 domains and exhibits activity in some cells, including myoblasts and endothelial cells. Recently, myoferlin was identified as a promising target and biomarker in non‐small‐cell lung cancer, breast cancer, pancreatic adenocarcinoma, hepatocellular carcinoma, colon cancer, melanoma, oropharyngeal squamous cell carcinoma, head and neck squamous cell carcinoma, clear cell renal cell carcinoma and endometrioid carcinoma. This evidence indicated that myoferlin was involved in the proliferation, invasion and migration of tumour cells, the mechanism of which mainly included promoting angiogenesis, vasculogenic mimicry, energy metabolism reprogramming, epithelial‐mesenchymal transition and modulating exosomes. The roles of myoferlin in both normal cells and cancer cells are of great significance to provide novel and efficient methods of tumour treatment. In this review, we summarize recent studies and findings of myoferlin and suggest that myoferlin is a novel potential candidate for clinical diagnosis and targeted cancer therapy.

## 1 BACKGROUND

Myoferlin is a relatively novel membrane‐anchored protein within the ferlin family. The myoferlin gene is located at chromosome 10q23.33. It was initially found to have a high degree of homology to dysferlin, and the percentage of similarity between myoferlin and dysferlin sequences is 69%.^1^ Dysferlin gene mutations cause Miyoshi myopathy and limb girdle muscular dystrophy type 2B,^2^ while mutations in myoferlin are not correlated with human disease in previous studies. However, myoferlin functions have been revealed through animal and cytology experiments. Elimination of myoferlin in mice results in smaller myofibres and a dystrophic phenotype that has a decreased capacity to regenerate after injury.^3^ In the hindlimb muscles of resistance exercise‐trained rats, myoferlin mRNA was highly up‐regulated.^4^ In quadriceps biopsies from Duchenne muscular dystrophy patients, myoferlin mRNA was also up‐regulated 7.3‐fold.^5^ Of note, recent studies suggest that myoferlin participates in the proliferation, invasion and metastasis of multiple cancers. In addition, researchers regard myoferlin as a promising target and biomarker. In this review, we will present a summary of the functions and roles of myoferlin in normal and tumour cells with updated knowledge.

## 2 THE STRUCTURE OF MYOFERLIN

**Figure 1:**
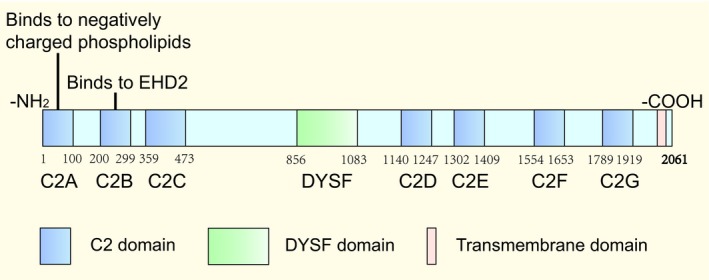
The structure of myoferlin. Myoferlin is a protein that is composed of 2061 amino acids and contains seven C2 domains (referred to C2A‐G), a DYSF domain and a carboxyl‐terminal transmembrane domain. C2A binds to negatively charged phospholipids; C2B binds to EHD2

Myoferlin belongs to the ferlin family, which has a single pass transmembrane domain situated at the carboxy‐terminus in common.^6^ The ferlin family is composed of five different proteins, namely, dysferlin, myoferlin, otoferlin, Fer1L5 and Fer1L6.^7^ All of the ferlin family proteins contain various C2 domains (C2B, C2C, C2D, C2E and C2F) and a carboxy‐terminal transmembrane domain. Dysferlin, myoferlin and Fer1L5 contain the C2A and DYSF domains. Otoferlin also contains the C2A domain.^7^ In the ferlin family, myoferlin shares more similarities with dysferlin compared with other ferlin proteins.^1^ Myoferlin and dysferlin are both 230 kD proteins that contain seven C2 domains and one carboxy‐terminal transmembrane domain that is a membrane‐spanning protein domain. Myoferlin and dysferlin both contain a DYSF domain and are highly expressed in myoblasts. An interesting feature of the DYSF structure is that it contains a DYSF domain within another DYSF domain due to gene duplication, but the function of the DYSF domain still remains unknown.^8^ Mature myofibres express dysferlin and myoferlin at the plasma membrane.^1^, ^3^ Furthermore, myoferlin contains some positively charged residues that are important for allowing the transmembrane domain to anchor within the membrane.^7^ Myoferlin is highly expressed in skeletal muscle, heart muscle and endothelial cells and is also expressed in lungs and most other tissues at low levels.^1^ Myoferlin typically works with the help of C2 domains. Each C2 domain is formed by approximately 100 amino acids. Two C2‐domains (C2A and C2B) are found in the cytoplasmic domain of synaptotagmin I. Synaptotagmin I works as a Ca^2+^ sensor that facilitates membrane fusion. C2 domains typically function by two mechanisms: binding phospholipids and playing roles in protein‐protein interactions.^9^ The C2 domain at the amino terminus of myoferlin, C2A, binds negatively charged phospholipids in response to calcium and is highly expressed in myoblasts undergoing fusion. The C2A domain of myoferlin only binds to phospholipid mixtures that have a high fraction of phosphatidylserine.^10^ This domain is ideally located to mediate vesicle trafficking directly.^11^ The second domain of myoferlin, C2B, binds to EH‐domain‐containing 2 (EHD2) directly. Thus, myoferlin is implicated in both endocytic‐recycling and clathrin‐mediated endocytosis.^12^ Of note, the C2D domain is a target of WJ460, a novel small molecule compound, which exhibits high therapeutic significance in various tumours.^13^ The structure of myoferlin is shown in Figure 1.

## 3 FUNCTIONS OF MYOFERLIN IN NORMAL CELLS

### 3.1 Muscle cells

**Figure 2:**
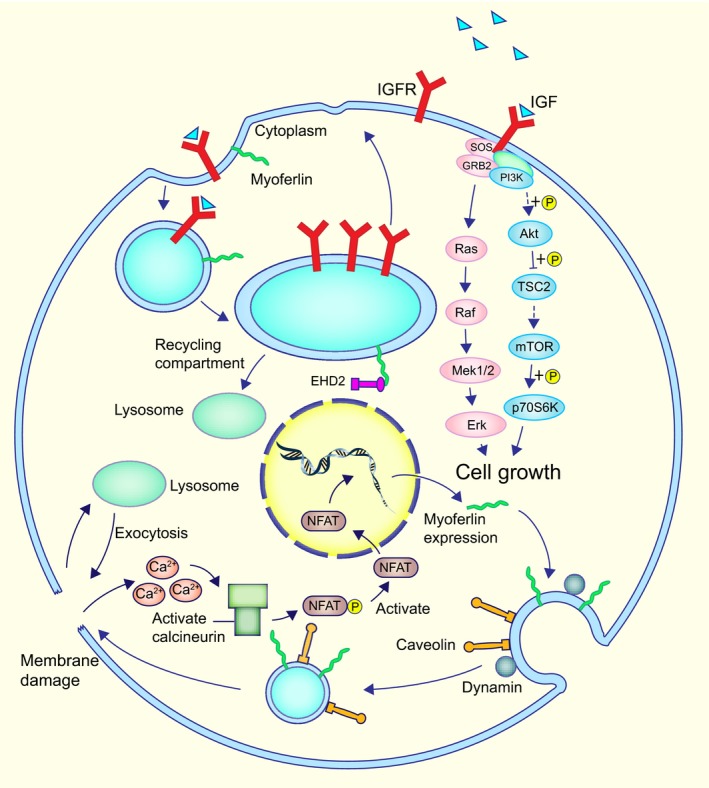
Models for myoferlin and EHD2 in vesicle cycling and myoferlin expression in damaged myofibres. Myoferlin and EHD2 are implicated in vesicle cycling. After endocytosis, some receptors and their ligands are internalized and then are shuttled to the endocytic‐recycling compartment. Finally, they are shuttled back to the membrane and work in another round. EHD2 binds directly to myoferlin in the endocytic‐recycling compartment and promotes the cyclic process of insulin‐like growth factor receptor (IGFR). IGF binds to IGFR directly to promote cell growth by activating the Akt/mTOR and MAPK pathway. In injured myofibres, Ca^2+^ flows into the cytoplasm and activates NFATs. NFAT then binds to the promoter of the myoferlin and increases the expression of myoferlin. Ca^2+^ influx is sensed by myoferlin and initiates dynamin‐dependent endocytosis, which cooperates with caveolin. The budded caveolae are then shuttled to the damaged area by an exocytic repair model

Skeletal muscle has inherent renewal properties that allow for regeneration and on‐going repair. The individual muscle cells are highly dependent on an effective and rapid vesicle trafficking system for efficient repair and growth.^14^ Vesicle trafficking system is crucial for many cellular functions, including cell division, cell migration and the regulation of signalling. In addition, the capacity to coordinate actin dynamics promoting cytoskeletal rearrangements plays an important role during myogenesis.^15^ Myoferlin functions in some of the above‐mentioned activities, and a summary of these mechanisms is presented in Figure 2.

#### 3.1.1 Essential for vesicle trafficking and muscle growth

Members of the EH‐domain‐containing (EHD) family act in the process of endocytosis and endocytic recycling, which mediate lipid and receptor recycling back to the plasma membrane.^16^ Myoferlin functions in this process as a critical participant with an asparagine‐proline‐phenylalanine motif in the myoferlin C2B domain and binds directly to EHD2, an EHD family molecule that is responsible for vesicular trafficking.

Insulin‐like growth factor (IGF) is an important protein in the regulation of muscle growth,^17^, ^18^ and its function is mediated by binding to the IGF1 receptor (IGF1R) then activating MAPK and AKT pathways.^19^ In developing myofibres, IGF1 stimulation induces muscle fibre hypertrophy.^14^ Myoferlin targets EHD2 to impact the translocation and recycling of IGF1R^20^ and is a modulator of muscle growth.^21^ In detail, myoferlin internalizes IGF1R and is subsequently shuttled back to the cell surface to bind more IGF1 in further rounds. In myoferlin‐null myoblasts, large vesicular structures are formed by the accumulation of IGF1R, and the receptors cannot be shuttled back to the membrane. In addition, EHD2 induces myotube formation,^15^ which is also affected by myoferlin.

#### 3.1.2 Mediating the fusion of myoblasts

Myoferlin is highly expressed for myoblast fusion.^11^ Myoferlin plays an important role in the formation of the myotubes by the fusion of singly nucleated myoblasts during the regeneration of mature muscle and embryonic muscle growth.^3^ Mechanically, it has been demonstrated that myoferlin is necessary for calcium‐sensitive membrane resealing via the C2A domain, which can promote the fusion of two opposed lipid bilayers. Furthermore, myoferlin interacts with EHD2 to regulate myoblast fusion by regulating reorganization or disassembly of the cytoskeleton^16^ given that the C2B domain of myoferlin can directly bind to EHD2 and compete with EHD2‐binding protein 1 (EHBP1), the binding partner of EHD2.^22^

#### 3.1.3 Promoting skeletal muscle repair

Skeletal muscle contains multinucleated myofibres, and myoferlin promotes their growth and repair by inducing mononucleated myoblasts to fuse with other mononucleated myoblasts and myoblasts to fuse to myotubes.^23^ Nuclear factor of activated T cells (NFAT) is expressed in muscle and works at different periods during the process of muscle growth and differentiation.^24^, ^25^, ^26^ NFAT targets a 1543‐bp fragment of the myoferlin promoter to induce the expression of myoferlin,^23^ which contributes significantly to the adaptation occurring in skeletal muscle during ground squirrel hibernation.^27^

### 3.2 Endothelial cells

#### 3.2.1 Positively regulating VEGFR‐2

Myoferlin is highly expressed in vascular tissues and endothelial cells (ECs), which are especially enriched in CEM/LR (caveolae‐enriched buoyant membrane microdomains/lipid raft) microdomains. Myoferlin functions in migration, proliferation and nitric oxide (NO) release, which occur in response to vascular endothelial growth factor (VEGF).^28^ In addition, myoferlin is necessary in some plasma membrane events, such as signalling in specialized cells. The loss of myoferlin reduces the autophosphorylation and expression of VEGF receptor‐2 (VEGFR‐2) in native ECs. The transfection of myoferlin increases autophosphorylation in response to VEGF and VEGFR‐2 membrane expression in a reconstituted cell system. A complex is formed by myoferlin, which also contains dynamin‐2 and VEGFR‐2. This protein complex is necessary for the surface expression of VEGFR‐2.^29^ This complex prevents proteasomal degradation and CBL‐dependent VEGFR‐2 polyubiquitination, which increases functional signalling and VEGFR‐2 protein stability.^28^ Myoferlin depletion disrupts rapid VEGF‐mediated intracellular signalling pathways and attenuates VEGF‐mediated activation of key intracellular signalling cascades, that is, c‐Jun N‐terminal kinase (JNK), phospholipase Cγ (PLCγ) and extracellular signal‐regulated kinase‐1/2 (ERK‐1/2).^30^

#### 3.2.2 Up‐regulating Tie‐2

Myoferlin is required for proper tyrosine kinase receptors expression at the plasma membrane. Myoferlin knockdown in ECs decreases the expression of a second tyrosine kinase receptor, Tie‐2, which is a well‐described angiogenic receptor.^31^ In addition, a study demonstrated that myoferlin gene silencing results in oedema formation and attenuates angiogenesis.^32^ Post‐translational regulation of many tyrosine kinase receptors requires myoferlin, including VEGFR‐2 and Tie‐2. Acute myoferlin knockdown may exhibit anti‐angiogenic effects and act as an anti‐angiogenesis target in the treatment of cancer or other angiogenesis‐related diseases.^32^

#### 3.2.3 Modulating receptor‐dependent endocytosis

Myoferlin induces endomembrane fusion with the plasma membrane in endothelial cells. Myoferlin also regulates aspects of receptor‐dependent endocytosis. Myoferlin gene silencing decreases caveolae/raft‐dependent endocytosis, whereas ectopic myoferlin expression increases endocytosis in COS‐7 cells.^29^ Mechanistically, myoferlin partially colocalizes with caveolin‐1 (Cav‐1) and dynamin‐2 (Dyn‐2) to form a protein complex, which participates in membrane fusion and caveolae‐dependent endocytosis.^29^ Current research defines this complex as a molecular bandage that may be essential to the integrity of the cellular membrane. The molecular bandage may also provide a method to regulate various disease processes.^33^

## 4 ROLES OF MYOFERLIN IN CANCERS

Recently, myoferlin has roles in various cancers, and current studies suggest that myoferlin is a promising target and biomarker. Myoferlin is involved in the proliferation,^34^ invasion and migration^35^ of cancer cells via different mechanisms, mainly including promoting angiogenesis,^36^ vasculogenic mimicry,^37^ energy metabolism reprogramming,^38^, ^39^ epithelial‐mesenchymal transition (EMT)^40^ and affecting exosomes.^41^ Clinically, myoferlin levels correlate with histologic grade and prognosis in several types of cancers. Of note, the functions and mechanisms of myoferlin in cancers have not been thoroughly revealed to date. As myoferlin is considered a highly potential therapeutic target, further exploration is required. Myoferlin expression in cancers has been recently assessed and is presented in Table 1 below.

**Table 1:** Expression of myoferlin in cancers

Cancer	Total no. of samples	Positive rate	Correlation of myoferlin level and tumour stage or grade	References
NSCLC	148	50.7%	Stage: No correlation observed (*P* = .632)	42
Breast cancer	90	No accurate data	Stage: Positive correlation	13
PAC	154	41.6%	Histologic grade: Positive correlation	43
HCC	138	No accurate data	Stage or grade: No correlation observed	44
Melanoma	52	42.3%	Pathological grade: No correlation observed (*P* = .190)	37
Colon cancer	28	76%	Prognostic or TNM stage: No correlation observed	38
OPSCC	211	78.2%	T stage: Positive correlation^a^	45
HNSCC	20	No accurate data	Not mentioned	46
ccRCC	304^b^	No accurate data	Fuhrman nuclear grade: Positive correlation	47
Endometrioid carcinoma	60	96.7%	FIGO stage: Negative correlation^c^	48

a Correlation between nuclear myoferlin expression and tumour stage.

b 304 samples were gathered from 152 patients.

c Correlation between myoferlin and FIGO stage or FIGO histologic grading showed a negative correlation (opposite to the results in other cancers), the mechanism of which had not been fully revealed (see Section 4.2.7).

### 4.1 Overview

#### 4.1.1 Promoting proliferation, invasion and migration

Unregulated proliferation is one of the major characteristics of tumour cells. Researchers observed that myoferlin protein is essential for the proliferation of breast cancer and pancreatic ductal adenocarcinoma (PDAC) cells.^34^, ^36^

Epithelial‐mesenchymal transition is a common phenomenon and a crucial step in the progression and dissemination of cancer metastasis.^49^ Functionally, myoferlin can induce EMT via up‐regulating mesenchymal cell markers, such as fibronectin and vimentin, and co‐ordinately down‐regulating epithelial markers, such as E‐cadherin.^35^, ^40^

A mathematical model prediction reveals that matrix metalloproteinases (MMPs) have a key function on cancer cell invasion.^50^ Myoferlin can also cause selective changes of MMPs in breast cancer^40^, ^50^ and melanoma.^37^

**Figure 3:**
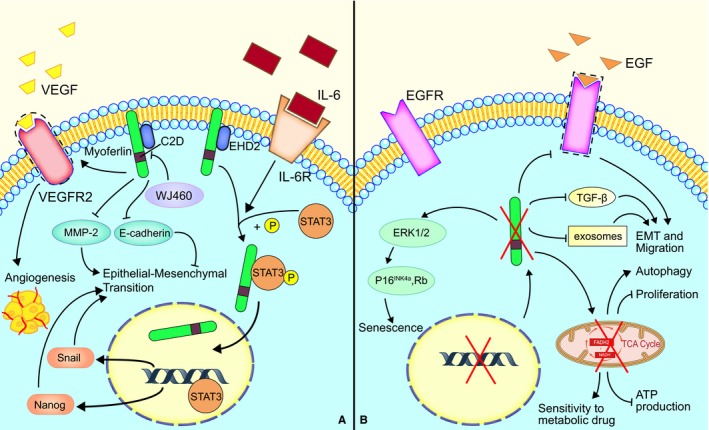
Myoferlin plays key roles in the development of cancer cells. A, Myoferlin is essential for the secretion of VEGFA and the function of VEGFR, which promotes angiogenesis and vasculogenic mimicry in cancer cells. Myoferlin has a significant function in IL‐6‐meditated tumour growth and tumour metastasis through promoting the nuclear translocation of STAT3. Furthermore, WJ460 binds to the C2D domain of myoferlin, which is considered a potential therapeutic target. B, Myoferlin depletion modulates MAPK and p16‐/Rb pathways and induces senescence. Myoferlin depletion decreases the capacity of exosomes and TGF‐β secretion, which has a negative function in EMT and migration. Myoferlin depletion also has an effect on endosomal and metabolism systems and inhibits cancer cell proliferation

A summary of these mechanisms is shown in Figure 3.

#### 4.1.2 Functions in exosomes

Exosomes, which are characterized by unique proteomic composition, participate in the intercellular exchange of metabolites, proteins and nucleic acids.^51^ Through proteomic analysis, myoferlin has been reported in several tumour‐derived exosomes, including prostate cancers,^52^ bladder cancers,^53^ colon cancers,^54^ ovary cancers,^55^ Hepatocellular carcinoma,^56^ squamous carcinoma cells^57^ and melanoma.^58^

Blomme et al^41^ revealed that myoferlin is an emerging oncogene that plays a functional role in exosome biology. Myoferlin is potentially involved in protein loading of exosome. The authors note that myoferlin is a general component of exosomes derived from pancreatic and breast cancer cell lines, and myoferlin‐depleted exosomes exhibit a reduced capacity to transfer nucleic acids to human endothelial cells, thus reducing the ability to induce proliferation and migration of human endothelial cells.

#### 4.1.3 Promoting angiogenesis/vasculogenic mimicry

**Figure 4:**
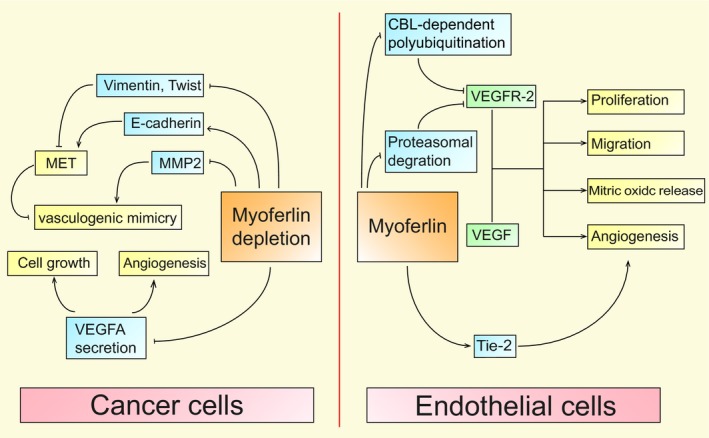
Myoferlin participates in angiogenesis in cancer and endothelial cells. In cancer cells, myoferlin depletion inhibits the secretion of VEGFA, which has a negative effect on proliferation and angiogenesis. Myoferlin depletion suppresses vasculogenic mimicry formation via down‐regulating MMP2 and induces the mesenchymal to epithelial transition (MET). In endothelial cells, myoferlin prevents proteasomal degradation and CBL‐dependent VEGFR‐2 polyubiquitination, which increases VEGFR‐2 protein stability and VEGF‐mediated intracellular signalling pathways. Myoferlin knockdown also down‐regulates the well‐described angiogenic receptor Tie‐2

Myoferlin gene knockdown attenuates the expression of VEGFR‐2 and Tie‐2, well‐described angiogenic receptors, in endothelial cells.^32^ However, in several cancer cells, myoferlin also has another role. A recent study reported that myoferlin overexpression induced the formation of vasculogenic mimicry in melanoma.^37^ Concrete mechanisms are discussed in the next section. The mechanism and the exact regulatory system are presented in Figure 4.

#### 4.1.4 Promoting energy metabolism reprogramming

Energy metabolism reprogramming is considered an emerging hallmark and a therapeutic target in cancers for cancer cells mostly prefer performed with glycolysis to produce energy.^59^ But cancer cells with more flexible metabolic phenotype have stronger resistance. In triple‐negative breast cancer,^60^ PDAC^39^ and colon cancer^38^ cells, myoferlin was described as an essential molecule to maintain a high oxidative phosphorylation activity. The mechanism will be discussed in the next section.

#### 4.1.5 Clinical significance

Myoferlin plays an oncogenic role and promotes cancer cell metastasis; thus, it often exhibits a relationship with histological grade or clinical stage of cancers. In addition, overexpression of myoferlin often indicates a poor prognosis. Specifically, myoferlin is an independent prognostic factor in pancreatic adenocarcinoma,^36^, ^43^ oropharyngeal squamous cell carcinoma,^45^ head and neck squamous cell carcinoma patients,^45^, ^61^ clear cell renal cell carcinoma,^47^ colon cancer^38^ and non‐small‐cell lung carcinoma^42^ patients.

### 4.2 Specific roles of myoferlin in different cancers

#### 4.2.1 Myoferlin in non‐small‐cell lung carcinoma

Lung cancer is a major cause of cancer‐related deaths worldwide.^62^ Non‐small‐cell lung cancer (NSCLC) accounts for approximately 85%‐90% of all lung cancers. Myoferlin is expressed in normal lung parenchyma and normal bronchial epithelium.^63^ A recent study found that myoferlin localizes to the cytoplasm of the cells in all NSCLC pathological subtypes, and adenocarcinomas exhibited the largest proportion.^42^ Moreover, in adenocarcinoma cases, myoferlin‐positive tumours may indicate a poor prognosis (odds ratio = 2.94; *P* = .339), while myoferlin and VEGFR‐2 expression exhibited a significant correlation in squamous cell carcinoma (*P* = .001), especially in stage I patients.^42^

#### 4.2.2 Myoferlin in breast cancer

Female breast cancer is the second commonly diagnosed cancer in the world and the leading cause of cancer associated death in females.^62^ Studies have shown that myoferlin plays a critical role in cancer progression via various mechanisms. For migration, researchers found that myoferlin depletion enhances cell adhesion, enlarges focal adhesion,^64^ enhances cell‐matrix adhesion through elevating focal adhesion kinase and paxillin phosphorylation,^65^ redirects cancer cell motility^35^ and subsequently inhibits migration.

In molecular level, potential targets of myoferlin include MMPs, EGFR and TGF‐β. After knocking‐down myoferlin, mesenchymal to epithelial transition occurred,^40^ and selective changes in MMPs were observed (which included remarkable down‐regulation of MMP1^40^, ^50^ and MMP3, 8, 12, 13, 14, 16^40^ and up‐regulation of MMP9^40^), EGF‐induced cell migration and EMT were blocked^66^ (verified by impaired degradation of phosphorylated EGFR via dysfunctional plasma membrane caveolae and alteration of caveolin homo‐oligomerization) and bio‐mechanical properties were altered^65^ (cell stiffness decreased, cell‐substrate adhesion increased, and cells subsequently migrated directionally, collectively and slowly). In addition, vesicle traffic was impaired^60^ (saturated/unsaturated fatty acids were subsequently imbalanced, mitochondrial dysfunction occurred, metabolic reprogramming to glycolysis was triggered, the ability to balance between oxidative phosphorylation and glycolysis was reduced, and the sensitivity to metabolic drug increased), growth velocity was reduced^34^ (partly for myoferlin‐dependent plasma membrane fusion and fission events were inhibited). Moreover, the overall migration ability of the tumour decreased^64^ (due to the abovementioned mechanisms, and reduced autocrine TGF‐β production caused dysregulation of TGF‐β1 signalling). Of note, the author stated that mechanism by which myoferlin regulates TGF‐β1 secretion may occur altered gene expression or exocytosis, which deserves further study.^64^ Of note, Zhang et al^13^ found that myoferlin was directly targeted by WJ460 and suggest that targeting myoferlin by WJ460 may be a promising therapeutic strategy in myoferlin‐driven breast cancers.

#### 4.2.3 Myoferlin in pancreatic adenocarcinoma

Pancreatic cancer is one of the deadliest cancers given its poor prognosis.^62^ Similarly, high myoferlin levels are a risk factor in pancreatic adenocarcinoma (PAC). Myoferlin expression significantly correlates with the degree of histological differentiation of PAC, and reduced myoferlin expression alleviated malignant phenotypes of both primary and metastatic PAC cells.^43^ Exosomes have various roles in PDAC, mainly including local invasion, migration, immune evasion and therapeutic resistance.^67^ Myoferlin is critical for producing functional exosomes with sufficient quantities of certain components.^41^

Autocrine and paracrine of vascular endothelial growth factor A (VEGFA) have pro‐proliferative effects independently.^20^, ^68^ Myoferlin knockdown down‐regulated VEGFA secretion, which is caused by impairment of VEGFA exocytosis, subsequently, tumours lacked VEGFA and VEGFA induced functional blood vessels thus exhibited a reduced volume.^36^ In addition, myoferlin is required to maintain a branched mitochondrial structure and high oxidative phosphorylation activity. Myoferlin depletion induces the phosphorylation of dynamin‐related protein (DRP)‐1 and increases its abundance, thus leading to mitochondrial fission and swelling.^39^ Interestingly, depletion of myoferlin led to a reduction in autophagy induction.^39^ Li et al reported lead compound 6y, one of 1,5‐diaryl‐1,2,4‐triazole derivatives, bound to myoferlin and inhibited pancreatic cancer metastasis. The anticancer activity of 6y is positively associated with the expression of myoferlin, and 6y is insensitive to myoferlin depletion. The specific mechanisms may be mediated by blocking the receptor tyrosine kinases, inhibiting the secretions of matrix metalloproteinase and reversing the EMT process.^69^ Overall, myoferlin may provide a novel therapeutic target in PAC.

#### 4.2.4 Myoferlin in hepatocellular carcinoma

In total, 75%‐85% of liver cancer cases are hepatocellular carcinoma (HCC).^62^ HCC is often associated with poor prognosis.^70^ Hermanns et al^44^ demonstrated that myoferlin was necessary for invasion, proliferation and anchorage‐independent cell growth of HCC, and the myoferlin gene is targeted by MKL1/2. Furthermore, myoferlin inhibits EGFR and the downstream MAPK and p16‐/Rb pathways, thus affecting senescence phenotype. In detail, depletion of myoferlin in tumour cells from SRF‐VP16‐derived murine HCCs induced a senescence phenotype, which suggested that myoferlin might be a novel therapeutic target.

#### 4.2.5 Myoferlin in colon cancer

Colorectal cancer ranks third in terms of incidence but second in terms of mortality worldwide,^62^ and the most common tumour location is the proximal colon (41%) in America.^71^ Myoferlin's functions are similar to those in PAC. Myoferlin is required for high oxidative phosphorylation activity and maintenance of an organized mitochondrial network.^38^ In addition, Rademaker et al^38^ also found that myoferlin silencing causes reactive oxygen species (ROS) accumulation, p53‐dependent reduction of cell growth, enhanced DNA damage response and increased apoptosis. These authors revealed that myoferlin could represent a suitable target for new anticancer therapies.

#### 4.2.6 Myoferlin in melanoma

Melanoma is the most aggressive type of skin cancer, and the appearance of vasculogenic mimicry (VM) in this type of cancer always indicates a poor prognosis.^72^ In VM, tumour cells mimic true vascular endothelium cells and form microvascular channels.^73^ Myoferlin knockdown significantly impaired the capability of A375 cells to form VM structures and subsequently inhibit cell invasion and migration in vitro. At the molecular level, down‐regulation of myoferlin decreases MMP‐2 expression and induces MET.^37^ Thus, myoferlin may represent a biomarker for VM formation and a risk factor for poor prognosis of melanoma patients.

#### 4.2.7 Myoferlin in other cancers

Kumar et al^45^ demonstrated that myoferlin could exist in the nucleus, cytosol or membrane of oropharyngeal squamous cell carcinoma (OPSCC) cells. These authors uncovered that nuclear myoferlin expression was directly associated with IL‐6 (*P* < .001), inversely associated with HPV status (*P* = .0014) and could predict poor clinical outcome in OPSCC patients independently. Yadav et al^46^ found that myoferlin is bound to EHD2 protein and modulates the IL‐6/STAT3 signalling pathway, which regulates the expression of IL‐6/STAT3 downstream genes, including snail and nanog, in head and neck squamous cell carcinoma (HNSCC) cell lines. Myoferlin knockdown significantly decreases tumour growth and metastasis of HNSCC. Song et al^47^ uncovered that high myoferlin levels correlate with unfavourable prognosis in clear cell renal cell carcinoma (ccRCC) patients. Amazingly, in endometrioid carcinoma, high expression of myoferlin was related to low‐grade carcinoma, while the loss of myoferlin expression was noted in high‐grade carcinoma, which was in contrast to findings in other cancers.^48^ The authors pointed out it was probably due to the fact that normal endometrial tissue underwent a continuous cycle of regeneration, in which myoferlin was implicated. As noncyclic continuous exposure to sex hormones contributes to the oncogenesis of endometrioid carcinoma, the authors predicted a potential correlation among cellular regeneration, hormonal effect and myoferlin expression.^48^ This finding may suggest that myoferlin plays a different role in endometrioid carcinoma, and the mechanism remains uncharacterized.

## 5 CONCLUSION AND PROSPECT

Myoferlin is a membrane‐anchored ferlin family protein. Myoferlin is highly expressed in skeletal muscle, heart muscle and endothelial cells and is essential for the growth, repair and normal function of cells as it modulates vesicle trafficking, cell fusion, receptor‐dependent endocytosis and the expression of certain receptors. Myoferlin is also expressed in most other tissues at low levels. However, in specific tumours, myoferlin is overexpressed and plays critical roles. Myoferlin functions in the proliferation, invasion and migration of cancer cells through various mechanisms. These findings identify myoferlin as a novel potential candidate for clinical diagnosis and targeted therapy.

The latest research shows that myoferlin regulates cancer‐derived exosomes and functions as a new player in exosome biology.^41^ As mentioned above, myoferlin impacts tumour‐associated angiogenesis by affecting VEGFA secretion and EGFR activity.^36^, ^66^ These findings suggest that myoferlin induces the malignant phenotype of cancers by altering the tumour microenvironment. Zhang et al^13^ found that WJ460 directly targets myoferlin, interacts with myoferlin C2 domain and hampers the proper function of myoferlin.^13^ WJ460 represents a potentially effective therapeutic molecule for preventing myoferlin‐related cancers and provides an opportunity for developing myoferlin‐targeted agents. Moreover, lead compound 6y, one of the 1,5‐diaryl‐1,2,4‐triazole derivatives, targets at myoferlin and prevents pancreatic cancer metastasis, which suggests 6y may also be a promising therapeutic strategy.^69^

## CONFLICT OF INTERESTS

The authors declare that they have no competing interests.

## AUTHORS' CONTRIBUTIONS

CR, WZ, BZ and CZ contributed to the study design. WZ, BZ and CZ drafted and critically revised the manuscript. CR, ZB, HX, XY, WL, BZ and LW discussed and revised the manuscript. All authors read and approved the final manuscript.

## ETHICS APPROVAL AND CONSENT TO PARTICIPATE

Not applicable.

## References

[1] Davis DB , Delmonte AJ , Ly CT , McNally EM . Myoferlin, a candidate gene and potential modifier of muscular dystrophy. Hum Mol Genet. 2000;9:217‐226. 10607832 10.1093/hmg/9.2.217

[2] Liu J , Aoki M , Illa I , et al. Dysferlin, a novel skeletal muscle gene, is mutated in Miyoshi myopathy and limb girdle muscular dystrophy. Nat Genet. 1998;20:31‐36. 9731526 10.1038/1682

[3] Doherty KR , Cave A , Davis DB , et al. Normal myoblast fusion requires myoferlin. Development. 2005;132:5565‐5575. 16280346 10.1242/dev.02155 PMC4066872

[4] Adams GR , Haddad F , Bodell PW , Tran PD , Baldwin KM . Combined isometric, concentric, and eccentric resistance exercise prevents unloading‐induced muscle atrophy in rats. J Appl Physiol (1985). 2007;103:1644‐1654. 17872405 10.1152/japplphysiol.00669.2007

[5] Haslett JN , Sanoudou D , Kho AT , et al. Gene expression comparison of biopsies from Duchenne muscular dystrophy (DMD) and normal skeletal muscle. Proc Natl Acad Sci USA. 2002;99:15000‐15005. 12415109 10.1073/pnas.192571199 PMC137534

[6] Jimenez JL , Bashir R . In silico functional and structural characterisation of ferlin proteins by mapping disease‐causing mutations and evolutionary information onto three‐dimensional models of their C2 domains. J Neurol Sci. 2007;260:114‐123. 17512949 10.1016/j.jns.2007.04.016

[7] Posey AD Jr , Demonbreun A , McNally EM . Ferlin proteins in myoblast fusion and muscle growth. Curr Top Dev Biol. 2011;96:203‐230. 21621072 10.1016/B978-0-12-385940-2.00008-5 PMC4464798

[8] Sula A , Cole AR , Yeats C , Orengo C , Keep NH . Crystal structures of the human Dysferlin inner DysF domain. BMC Struct Biol. 2014;14:3. 24438169 10.1186/1472-6807-14-3 PMC3898210

[9] Hui E , Bai J , Chapman ER . Ca2+‐triggered simultaneous membrane penetration of the tandem C2‐domains of synaptotagmin I. Biophys J. 2006;91:1767‐1777. 16782782 10.1529/biophysj.105.080325 PMC1544279

[10] Harsini FM , Bui AA , Rice AM , et al. Structural basis for the distinct membrane binding activity of the homologous C2A domains of myoferlin and dysferlin. J Mol Biol. 2019;431:2112‐2126. 31004665 10.1016/j.jmb.2019.04.006 PMC6599597

[11] Davis DB , Doherty KR , Delmonte AJ , McNally EM . Calcium‐sensitive phospholipid binding properties of normal and mutant ferlin C2 domains. J Biol Chem. 2002;277:22883‐22888. 11959863 10.1074/jbc.M201858200

[12] Grant BD , Caplan S . Mechanisms of EHD/RME‐1 protein function in endocytic transport. Traffic. 2008;9:2043‐2052. 18801062 10.1111/j.1600-0854.2008.00834.x PMC2766864

[13] Zhang T , Li J , He Y , et al. A small molecule targeting myoferlin exerts promising anti‐tumor effects on breast cancer. Nat Commun. 2018;9:3726. 30213946 10.1038/s41467-018-06179-0 PMC6137146

[14] Grounds MD , Shavlakadze T . Growing muscle has different sarcolemmal properties from adult muscle: a proposal with scientific and clinical implications: reasons to reassess skeletal muscle molecular dynamics, cellular responses and suitability of experimental models of muscle disorders. BioEssays. 2011;33:458‐468. 21500235 10.1002/bies.201000136

[15] Posey AD , Swanson KE , Alvarez MG , et al. EHD1 mediates vesicle trafficking required for normal muscle growth and transverse tubule development. Dev Biol. 2014;387:179‐190. 24440153 10.1016/j.ydbio.2014.01.004 PMC3987670

[16] Naslavsky N , Caplan S . EHD proteins: key conductors of endocytic transport. Trends Cell Biol. 2011;21:122‐131. 21067929 10.1016/j.tcb.2010.10.003 PMC3052690

[17] Yang SY , Goldspink G . Different roles of the IGF‐I Ec peptide (MGF) and mature IGF‐I in myoblast proliferation and differentiation. FEBS Lett. 2002;522:156‐160. 12095637 10.1016/s0014-5793(02)02918-6

[18] Musarò A , McCullagh K , Paul A , et al. Localized Igf‐1 transgene expression sustains hypertrophy and regeneration in senescent skeletal muscle. Nat Genet. 2001;27:195‐200. 11175789 10.1038/84839

[19] Rommel C , Bodine SC , Clarke BA , et al. Mediation of IGF‐1‐induced skeletal myotube hypertrophy by PI(3)K/Akt/mTOR and PI(3)K/Akt/GSK3 pathways. Nat Cell Biol. 2001;3:1009‐1013. 11715022 10.1038/ncb1101-1009

[20] Doherty KR , Demonbreun AR , Wallace GQ , et al. The endocytic recycling protein EHD2 interacts with myoferlin to regulate myoblast fusion. J Biol Chem. 2008;283:20252‐20260. 18502764 10.1074/jbc.M802306200 PMC2459265

[21] Demonbreun AR , Posey AD , Heretis K , et al. Myoferlin is required for insulin‐like growth factor response and muscle growth. FASEB J. 2010;24:1284‐1295. 20008164 10.1096/fj.09-136309 PMC2845429

[22] Guilherme A , Soriano NA , Bose S , et al. EHD2 and the novel EH domain binding protein EHBP1 couple endocytosis to the actin cytoskeleton. J Biol Chem. 2004;279:10593‐10605. 14676205 10.1074/jbc.M307702200

[23] Demonbreun AR , Lapidos KA , Heretis K , et al. Myoferlin regulation by NFAT in muscle injury, regeneration and repair. J Cell Sci. 2010;123:2413‐2422. 20571050 10.1242/jcs.065375 PMC2894657

[24] Abbott KL , Friday BB , Thaloor D , Murphy TJ , Pavlath GK . Activation and cellular localization of the cyclosporine A‐sensitive transcription factor NF‐AT in skeletal muscle cells. Mol Biol Cell. 1998;9:2905‐2916. 9763451 10.1091/mbc.9.10.2905 PMC25565

[25] Cho Y‐Y , Yao KE , Bode AM , et al. RSK2 mediates muscle cell differentiation through regulation of NFAT3. J Biol Chem. 2007;282:8380‐8392. 17213202 10.1074/jbc.M611322200 PMC2824544

[26] O'Connor RS , Mills ST , Jones KA , Ho SN , Pavlath GK . A combinatorial role for NFAT5 in both myoblast migration and differentiation during skeletal muscle myogenesis. J Cell Sci. 2007;120:149‐159. 17164296 10.1242/jcs.03307

[27] Zhang Y , Storey KB . Expression of nuclear factor of activated T cells (NFAT) and downstream muscle‐specific proteins in ground squirrel skeletal and heart muscle during hibernation. Mol Cell Biochem. 2016;412:27‐40. 26597853 10.1007/s11010-015-2605-x

[28] Bernatchez PN , Acevedo L , Fernandez‐Hernando C , et al. Myoferlin regulates vascular endothelial growth factor receptor‐2 stability and function. J Biol Chem. 2007;282:30745‐30753. 17702744 10.1074/jbc.M704798200

[29] Bernatchez PN , Sharma A , Kodaman P , Sessa WC . Myoferlin is critical for endocytosis in endothelial cells. Am J Physiol Cell Physiol. 2009;297:C484‐C492. 19494235 10.1152/ajpcell.00498.2008 PMC2740391

[30] Bernatchez PN , Allen BG , Gélinas DS , Guillemette G , Sirois MG . Regulation of VEGF‐induced endothelial cell PAF synthesis: role of p42/44 MAPK, p38 MAPK and PI3K pathways. Br J Pharmacol. 2001;134:1253‐1262. 11704645 10.1038/sj.bjp.0704367 PMC1573057

[31] Pan X , Liang L , Si RU , et al. Discovery of novel anti‐angiogenesis agents. Part 10: multi‐target inhibitors of VEGFR‐2, Tie‐2 and EphB4 incorporated with 1,2,3‐triazol. Eur J Med Chem. 2019;163:1‐9. 30503935 10.1016/j.ejmech.2018.11.042

[32] Yu C , Sharma A , Trane A , Utokaparch S , Leung C , Bernatchez P . Myoferlin gene silencing decreases Tie‐2 expression in vitro and angiogenesis in vivo. Vascul Pharmacol. 2011;55:26‐33. 21586340 10.1016/j.vph.2011.04.001

[33] Cipta S , Patel HH . Molecular bandages: inside‐out, outside‐in repair of cellular membranes. Focus on "Myoferlin is critical for endocytosis in endothelial cells". Am J Physiol Cell Physiol. 2009;297:C481‐C483. 19587215 10.1152/ajpcell.00288.2009 PMC2740387

[34] Leung C , Yu C , Lin MI , Tognon C , Bernatchez P . Expression of myoferlin in human and murine carcinoma tumors: role in membrane repair, cell proliferation, and tumorigenesis. Am J Pathol. 2013;182:1900‐1909. 23499551 10.1016/j.ajpath.2013.01.041

[35] Volakis LI , Li R , Ackerman WE , et al. Loss of myoferlin redirects breast cancer cell motility towards collective migration. PLoS ONE. 2014;9:e86110. 24586247 10.1371/journal.pone.0086110 PMC3935829

[36] Fahmy K , Gonzalez A , Arafa M , et al. Myoferlin plays a key role in VEGFA secretion and impacts tumor‐associated angiogenesis in human pancreas cancer. Int J Cancer. 2016;138:652‐663. 26311411 10.1002/ijc.29820

[37] Zhang W , Zhou P , Meng A , et al. Down‐regulating myoferlin inhibits the vasculogenic mimicry of melanoma via decreasing MMP‐2 and inducing mesenchymal‐to‐epithelial transition. J Cell Mol Med. 2018;22:1743‐1754. 29164766 10.1111/jcmm.13455 PMC5824422

[38] Rademaker G , Costanza B , Bellier J , et al. Human colon cancer cells highly express myoferlin to maintain a fit mitochondrial network and escape p53‐driven apoptosis. Oncogenesis. 2019;8:21. 30850580 10.1038/s41389-019-0130-6 PMC6408501

[39] Rademaker G , Hennequière V , Brohée L , et al. Myoferlin controls mitochondrial structure and activity in pancreatic ductal adenocarcinoma, and affects tumor aggressiveness. Oncogene. 2018;37:4398‐4412. 29720728 10.1038/s41388-018-0287-z PMC6085282

[40] Li R , Ackerman WE , Mihai C , Volakis LI , Ghadiali S , Kniss DA . Myoferlin depletion in breast cancer cells promotes mesenchymal to epithelial shape change and stalls invasion. PLoS ONE. 2012;7:e39766. 22761893 10.1371/journal.pone.0039766 PMC3384637

[41] Blomme A , Fahmy K , Peulen O , et al. Myoferlin is a novel exosomal protein and functional regulator of cancer‐derived exosomes. Oncotarget. 2016;7:83669‐83683. 27845903 10.18632/oncotarget.13276 PMC5347796

[42] Song DH , Ko GH , Lee JH , et al. Myoferlin expression in non‐small cell lung cancer: prognostic role and correlation with VEGFR‐2 expression. Oncol Lett. 2016;11:998‐1006. 26893682 10.3892/ol.2015.3988 PMC4734036

[43] Wang W‐S , Liu X‐H , Liu L‐X , et al. iTRAQ‐based quantitative proteomics reveals myoferlin as a novel prognostic predictor in pancreatic adenocarcinoma. J Proteomics. 2013;91:453‐465. 23851313 10.1016/j.jprot.2013.06.032

[44] Hermanns C , Hampl V , Holzer K , et al. The novel MKL target gene myoferlin modulates expansion and senescence of hepatocellular carcinoma. Oncogene. 2017;36:3464‐3476. 28114277 10.1038/onc.2016.496

[45] Kumar B , Brown NV , Swanson BJ , et al. High expression of myoferlin is associated with poor outcome in oropharyngeal squamous cell carcinoma patients and is inversely associated with HPV‐status. Oncotarget. 2016;7:18665‐18677. 26919244 10.18632/oncotarget.7625 PMC4951318

[46] Yadav A , Kumar B , Lang JC , Teknos TN , Kumar P . A muscle‐specific protein 'myoferlin' modulates IL‐6/STAT3 signaling by chaperoning activated STAT3 to nucleus. Oncogene. 2017;36:6374‐6382. 28745314 10.1038/onc.2017.245 PMC5690845

[47] Song DH , Ko GH , Lee JH , et al. Prognostic role of myoferlin expression in patients with clear cell renal cell carcinoma. Oncotarget. 2017;8:89033‐89039. 29179496 10.18632/oncotarget.21645 PMC5687666

[48] Kim MH , Song DH , Ko GH , et al. Myoferlin expression and its correlation with FIGO histologic grading in early‐stage endometrioid carcinoma. J Pathol Transl Med. 2018;52:93‐97. 29554794 10.4132/jptm.2017.11.29 PMC5859243

[49] Mao X‐Y , Li Q‐Q , Gao Y‐F , Zhou H‐H , Liu Z‐Q , Jin W‐L . Gap junction as an intercellular glue: emerging roles in cancer EMT and metastasis. Cancer Lett. 2016;381:133‐137. 27490999 10.1016/j.canlet.2016.07.037

[50] Eisenberg MC , Kim Y , Li R , Ackerman WE , Kniss DA , Friedman A . Mechanistic modeling of the effects of myoferlin on tumor cell invasion. Proc Natl Acad Sci U S A. 2011;108:20078‐20083. 22135466 10.1073/pnas.1116327108 PMC3250187

[51] Hu Y , Rao S‐S , Wang Z‐X , et al. Exosomes from human umbilical cord blood accelerate cutaneous wound healing through miR‐21‐3p‐mediated promotion of angiogenesis and fibroblast function. Theranostics. 2018;8:169‐184. 29290800 10.7150/thno.21234 PMC5743467

[52] Kharaziha P , Chioureas D , Rutishauser D , et al. Molecular profiling of prostate cancer derived exosomes may reveal a predictive signature for response to docetaxel. Oncotarget. 2015;6:21740‐21754. 25844599 10.18632/oncotarget.3226 PMC4673300

[53] Welton JL , Khanna S , Giles PJ , et al. Proteomics analysis of bladder cancer exosomes. Mol Cell Proteomics. 2010;9:1324‐1338. 20224111 10.1074/mcp.M000063-MCP201 PMC2877990

[54] Demory Beckler M , Higginbotham JN , Franklin JL , et al. Proteomic analysis of exosomes from mutant KRAS colon cancer cells identifies intercellular transfer of mutant KRAS. Mol Cell Proteomics. 2013;12:343‐355. 23161513 10.1074/mcp.M112.022806 PMC3567858

[55] Liang B , Peng P , Chen S , et al. Characterization and proteomic analysis of ovarian cancer‐derived exosomes. J Proteomics. 2013;80:171‐182. 23333927 10.1016/j.jprot.2012.12.029

[56] He M , Qin H , Poon T , et al. Hepatocellular carcinoma‐derived exosomes promote motility of immortalized hepatocyte through transfer of oncogenic proteins and RNAs. Carcinogenesis. 2015;36:1008‐1018. 26054723 10.1093/carcin/bgv081

[57] Park JE , Tan HS , Datta A , et al. Hypoxic tumor cell modulates its microenvironment to enhance angiogenic and metastatic potential by secretion of proteins and exosomes. Mol Cell Proteomics. 2010;9:1085‐1099. 20124223 10.1074/mcp.M900381-MCP200 PMC2877972

[58] Lazar I , Clement E , Ducoux‐Petit M , et al. Proteome characterization of melanoma exosomes reveals a specific signature for metastatic cell lines. Pigment Cell Melanoma Res. 2015;28:464‐475. 25950383 10.1111/pcmr.12380

[59] Yuan LW , Yamashita H , Seto Y . Glucose metabolism in gastric cancer: the cutting‐edge. World J Gastroenterol. 2016;22:2046‐2059. 26877609 10.3748/wjg.v22.i6.2046 PMC4726677

[60] Blomme A , Costanza B , de Tullio P , et al. Myoferlin regulates cellular lipid metabolism and promotes metastases in triple‐negative breast cancer. Oncogene. 2017;36:2116‐2130. 27775075 10.1038/onc.2016.369

[61] Yadav A , Kumar B , Datta J , Teknos TN , Kumar P . IL‐6 promotes head and neck tumor metastasis by inducing epithelial‐mesenchymal transition via the JAK‐STAT3‐SNAIL signaling pathway. Mol Cancer Res. 2011;9:1658‐1667. 21976712 10.1158/1541-7786.MCR-11-0271 PMC3243808

[62] Bray F , Ferlay J , Soerjomataram I , Siegel RL , Torre LA , Jemal A . Global cancer statistics 2018: GLOBOCAN estimates of incidence and mortality worldwide for 36 cancers in 185 countries. CA Cancer J Clin. 2018;68:394‐424. 30207593 10.3322/caac.21492

[63] Leung C , Shaheen F , Bernatchez P , Hackett TL . Expression of myoferlin in human airway epithelium and its role in cell adhesion and zonula occludens‐1 expression. PLoS ONE. 2012;7:e40478. 22808170 10.1371/journal.pone.0040478 PMC3393691

[64] Barnhouse VR , Weist JL , Shukla VC , et al. Myoferlin regulates epithelial cancer cell plasticity and migration through autocrine TGF‐beta1 signaling. Oncotarget. 2018;9:19209‐19222. 29721195 10.18632/oncotarget.24971 PMC5922389

[65] Blackstone BN , Li R , Ackerman WE , Ghadiali SN , Powell HM , Kniss DA . Myoferlin depletion elevates focal adhesion kinase and paxillin phosphorylation and enhances cell‐matrix adhesion in breast cancer cells. Am J Physiol Cell Physiol. 2015;308:C642‐C649. 25631868 10.1152/ajpcell.00276.2014

[66] Turtoi A , Blomme A , Bellahcene A , et al. Myoferlin is a key regulator of EGFR activity in breast cancer. Cancer Res. 2013;73:5438‐5448. 23864327 10.1158/0008-5472.CAN-13-1142

[67] Massoumi RL , Hines OJ , Eibl G , King JC . Emerging evidence for the clinical relevance of pancreatic cancer exosomes. Pancreas. 2019;48:1‐8. 30531240 10.1097/MPA.0000000000001203

[68] von Marschall Z , Cramer T , Höcker M , et al. De novo expression of vascular endothelial growth factor in human pancreatic cancer: evidence for an autocrine mitogenic loop. Gastroenterology. 2000;119:1358‐1372. 11054395 10.1053/gast.2000.19578

[69] Li Y , He Y , Shao T , et al. Modification and biological evaluation of a series of 1,5‐diaryl‐1,2,4‐triazole compounds as novel agents against pancreatic cancer metastasis through targeting myoferlin. J Med Chem. 2019;62:4949‐4966. 31026162 10.1021/acs.jmedchem.9b00059

[70] Yang YU , Wen F , Li J , et al. A high baseline HBV load and antiviral therapy affect the survival of patients with advanced HBV‐related HCC treated with sorafenib. Liver Int. 2015;35:2147‐2154. 25676812 10.1111/liv.12805

[71] Siegel RL , Miller KD , Fedewa SA , et al. Colorectal cancer statistics, 2017. CA Cancer J Clin. 2017;67:177‐193. 28248415 10.3322/caac.21395

[72] Yang JP , Liao YD , Mai DM , et al. Tumor vasculogenic mimicry predicts poor prognosis in cancer patients: a meta‐analysis. Angiogenesis. 2016;19:191‐200. 26899730 10.1007/s10456-016-9500-2

[73] Huang B , Xiao E , Huang M . MEK/ERK pathway is positively involved in hypoxia‐induced vasculogenic mimicry formation in hepatocellular carcinoma which is regulated negatively by protein kinase A. Med Oncol. 2015;32:408. 25487444 10.1007/s12032-014-0408-7

